# Serious psychological distress and disability among older persons living in conflict affected areas in eastern Ukraine: a cluster-randomized cross-sectional household survey

**DOI:** 10.1186/s13031-019-0194-0

**Published:** 2019-03-25

**Authors:** Aimee Summers, Eva Leidman, Isabel Maria Pereira Figueira Periquito, Oleg O. Bilukha

**Affiliations:** 1 Emergency Response and Recovery Branch, Division of Global Health Protection, Center for Global Health, Centers for Disease Control and Prevention, 1600 Clifton Road, MS E-22, Atlanta, 30329 USA; 2 The World Health Organization; Yemen Country Office, Nutrition Department, Algeria Street, Sana’a, Yemen

**Keywords:** Mental health, Warfare, Conflict, Elderly, Disability

## Abstract

**Background:**

Older persons are often unable to leave conflict areas; however, little is known about the mental and physical health among this population. Our objective was to determine the prevalence of and whether there was an association between psychological distress and disability among older persons affected by conflict in eastern Ukraine.

**Methods:**

We conducted a cluster-randomized cross-sectional household survey of persons aged ≥60 years in government and non-government controlled areas (GCA and NGCA) of Donetsk and Luhansk regions in January–March 2016. Psychological distress and dependency (degree of disability) were measured using the Kessler K6 Psychological Distress Scale and Katz Index of Independence in Activities of Daily Living, respectively. Association between psychological distress and dependency was assessed using logistic regression adjusting for demographic and socioeconomic characteristics.

**Results:**

Final sample included 758 and 418 persons in GCA and NGCA, respectively. Prevalence of serious psychological distress was 33.6% (95% Confidence Interval (CI), 28.0**–**39.7%) in GCA and 42.5% (95%CI, 36.1**–**49.2%) in NGCA. Overall, 32.2% (95%CI, 27.9**–**36.7%) of independent persons and 74.0% (95%CI, 65.2**–**81.2%) of moderately/severely dependent persons reported serious psychological distress (*P* < .0001). Being dependent, a woman, and having a chronic disease were all significantly associated with psychological distress in a logistic regression model.

**Conclusions:**

Prevalence of serious psychological distress was very high compared with rates reported from developed countries and was highly associated with disability. Health services for the disabled, including psychological as well as physical support, could help in reducing the proportion of people needing mental health services not normally identified.

**Electronic supplementary material:**

The online version of this article (10.1186/s13031-019-0194-0) contains supplementary material, which is available to authorized users.

## Background

During humanitarian emergencies, older persons are among the most vulnerable groups because of their often lower socioeconomic status and limited physical capabilities compared with younger adults [1]. Despite this vulnerability, they have not traditionally been considered a priority group for humanitarian assistance [1]. The United Nations High Commissioner for Refugees (UNHCR) policy on older refugees has highlighted three major problems they may face: social disintegration, negative social selection, and chronic dependency [2]. Social disintegration occurs when formal or informal social support erodes in response to war, flight, economic or security pressures, which cause separation and dispersal of families [2]. Negative social selection refers to the ability of the healthy and able-bodied to depart, leaving behind weaker and more vulnerable members of the group, including older persons [2]. Older persons often have disrupted social support networks with no one to care for them, in addition to reduced spending power because of low pensions and the inability to work [2]. Chronic dependency is dependency on other persons resulting from morbidity, such as non-communicable diseases, injuries, loss of hearing and eyesight, and loss of mobility [2]. Globally the proportion of older persons among the population is growing faster than any other age group. By 2050, more people worldwide will be > 60 years old than < 12 years old [1]. Persons > 60 years of age comprise 9% of the overall refugee population and in some places over 30% [1]. In 2016, 22% of Ukraine’s population was ≥60 years old [3].

Older persons are known to have higher rates of disability in the general population, adding to their vulnerability [4, 5]. Persons with disabilities are one of the most socially excluded groups in crisis-affected communities [1]. These persons face increased risk of discrimination, exploitation, and violence and additional barriers to accessing services and assistance [1]. Little is known about the mental and physical health of older persons in emergency and conflict situations, and even less is known about older persons with disability in these situations. Older persons with disabilities may experience increased psychological distress [2].

Ukraine was considered a stable, middle-income country prior to hostilities, which began in February 2014 [6]. These hostilities led to the division of Donetsk and Luhansk regions in eastern Ukraine into areas controlled by the government of Ukraine (GCA) and by separatist groups [non-government controlled areas (NGCA)] [6]. Persons living in the NGCA are more isolated compared with persons in GCA. They must cross the contact line which separates the two areas in order to collect their pensions and other social benefits and have their identification documents issued [7]. Persons living in GCA have greater access to health facilities and markets [7]. As of February 2016, nearly 10,000 civilians had been killed during the fighting between Ukrainian government forces and separatist groups [8]. Shelling forced some families to seek shelter in basements for weeks and others to leave the area. Basic services such as water, electricity, and health care were interrupted [8]. There were a total of 1.5 million internally displaced persons (IDPs) in Ukraine and over three million people affected by the conflict in February 2016 [8]. Over 60% of the displaced population were older persons [9]. International humanitarian actors have provided mobile health clinics in some areas, however there are very limited psychosocial services available [10]. An assessment conducted by HelpAge International in 2015 found that older persons in Donetsk and Luhansk in both GCA and NGCA experienced serious protection concerns, mostly linked to limited access to basic services and psychological distress [11]. It also reported that many older persons with disabilities or a chronic medical condition described lack of at least one of the following: humanitarian aid, social services, or health care [11]. Given the unknown prevalence of disability and psychological distress in older persons, and the reported lack of services, this population was of particular concern. This study aimed to determine the prevalence of psychological distress and disability among older persons affected by conflict in eastern Ukraine, whether there was an association between those conditions, and to compare the prevalence between persons living in GCA and NGCA.

## Methods

We conducted a cluster-randomized cross-sectional household survey of all persons aged ≥60 years in GCA and NGCA conflict affected areas of Donetsk region and GCA of Luhansk region in eastern Ukraine. Persons were eligible if they were a current resident of a randomly selected cluster and household and consented to participate. The total sample size of 743 persons in GCA was determined based on an expected 60% prevalence of hypertension, a ± 4.5% precision, and a design effect of 1.5. The sample size for NGCA of 418 persons was based on 60% prevalence of hypertension, a ± 6% precision, and a design effect of 1.5. The increased precision was required for GCA in order to allow for disaggregation by region during analysis.

The sample frame for selection was obtained from the Central Election Commission of Ukraine using data updated in October 2014; electoral precincts were used as the primary sampling units (clusters). All districts in Donetsk region were included. Five districts of Luhansk region were excluded because of their remoteness from the conflict zone. Clusters were selected probability proportional to size. In GCA, 30 clusters and four reserve clusters were selected; in NGCA 25 clusters and three reserve clusters were selected. Households for the second stage of sampling were systematically selected from random starting points in each cluster.

The survey included questions on household and individual characteristics, income, access to humanitarian assistance, chronic diseases and access to medications, acute conditions and health seeking behaviors, food intake and food security, dependency, and psychological distress (Additional file 1: Assessment Questionnaire, Ukraine 2016). Chronic diseases included in the questionnaire were: hypertension, diabetes, cardiac or vascular diseases, and chronic lung disease; all respondents self-reported whether a medical professional had diagnosed them with any of the chronic diseases listed. Persons self-reporting diagnosis of at least one of the included chronic conditions were categorized as having any chronic disease. This paper focuses on dependency and psychological distress.

Katz Index of Independence in Activities of Daily Living, a commonly used instrument for assessment, was used to assess dependency [12]. This scale includes questions on six activities of daily living: needing help to bathe, get dressed, use the toilet, move from bed to chair, eat, and whether the person is incontinent. The scale classifies persons as moderately dependent if they need help with two or three activities and as severely dependent if help is needed for ≥ four activities. In addition, we asked if someone was available to assist if the respondent needed assistance with one or more activity. This is an additional question not included in the Katz Index, but recommended in the Rapid Assessment Method for Older People to measure unmet need [13].

Psychological distress was measured using the Kessler K6 Psychological Distress Scale, which was previously validated in Ukraine and includes six questions about depressive and anxiety symptoms [14, 15]. Respondents were asked to self-report how frequently they felt nervous, hopeless, restless or fidgety, depressed, that everything was an effort, and worthless in the 30 days preceding the survey. Total scores range from six (indicating no distress) to 24 (indicating severe distress). Scores of > 12 indicate serious psychological distress. The Kessler scale has demonstrated excellent internal consistency and reliability (Cronbach’s alpha = 0.89) [14]. Pre-existing psychological distress prior to the 30 days preceding the survey was not assessed.

The questionnaire was developed in English and translated into Russian with accuracy checked by Russian-speaking staff from the United States Centers for Disease Control and Prevention (CDC). Enumerators located in eastern Ukraine experienced in conducting household surveys administered the questionnaires. If persons were incoherent and could not answer for themselves, the Kessler scale was not administered, however their caregiver answered questions related to demographics and the Katz Index. Enumerators were trained by Russian-speaking CDC staff on sampling procedures, questionnaire administration, and interview techniques. Prior to the start of data collection, a field test was conducted with older persons ineligible for the survey.

Data were entered by trained staff. Double data entry was performed and any discrepancies were reconciled using the paper forms. Analyses were conducted using STATA v13 (College Station, TX). Frequency of each indicator was calculated and adjusted to account for the complex survey design and combined analyses were weighted proportionally. *P*-values were calculated using two-proportion t-tests to assess differences between GCA and NGCA. Difference in median age and Kessler score between the two areas was tested using a two-sample Wilcoxon rank-sum test. Cronbach’s alpha was calculated to determine the reliability of the Kessler scale in our study sample.

The association of potential risk factors with serious psychological distress as the outcome variable were assessed using bivariate and multivariate logistic regression models. Potential risk factors used as predictor variables were: living in GCA or NGCA, age, household income per capita, education, sex, whether the person had been diagnosed with a chronic disease, whether the person was living alone, whether the respondent owned or rented their current place of residence, and dependency (moderate or severe). Statistically significant risk factors at a *P* ≤ 0.05 level were included in a multivariate logistic regression model to estimate odds ratios (OR) and 95% confidence intervals (CI). Data presented in the results are from the multivariate models and only include those variables that remained statistically significant in the multivariate regression model. All variables apart from household income had a < 1% missing rate (14% of household income variables were missing), because of the low rate of missing variables, persons with missing data were excluded from analyses only for variables for which data were missing. Persons who were incoherent or could not answer for themselves and to whom the Kessler scale was not administered were not included in the psychological distress analyses.

Prior to survey administration, persons read or the survey teams read aloud, a consent form detailing the purpose and risks and benefits of the study, how their household was selected, the procedures through which their information would be kept confidential, and that they could choose to not answer any questions and to stop the survey at any time. All persons provided verbal consent before participating in the survey. This study was determined to be non-research by the CDC Institutional Review Board.

## Results

The household survey was conducted in GCA January 30–February 13, 2016 and in NGCA February 21–March 5, 2016. In GCA, three of the originally selected clusters were inaccessible because of security and were replaced with four reserve clusters. The final sample included 31 clusters (24 in Donetsk region and seven in Luhansk region). In NGCA, all 25 clusters were accessible. Table 1 shows the eligibility of households and response rate for eligible households and respondents among persons ≥60 years old. In GCA, a total of 2525 households were visited. Of these, 671 (26.6%) included at least one older person who consented to participate in the survey. Eight hundred eighty-six older persons lived in the 671 consenting households and of these 758 (85.2%) adults agreed to be interviewed. In NGCA, 1324 households were visited; 310 households (23.4%) included at least one older person who consented to participate in the survey and 418 of 423 (98.8%) adults who lived in the consenting households agreed to be interviewed. About 7% of respondents in both areas were unable to answer for themselves and were not included in the mental health assessment.

**Table 1:** Eligibility of households and response rate for eligible households and respondents, Ukraine, 2016

Response	GCA n (%)	NGCA n (%)
Total Households	*N* = 2525	*N* = 1324
Absent or abandoned	1164 (46.1)	701 (52.9)
Refused	109 (4.3)	14 (1.1)
No one over 60	581 (23.0)	299 (22.6)
Consented	671 (26.6)	310 (23.4)
Total Respondents over 60 in Consenting Households	*N* = 890	*N* = 423
Absent	52 (5.8)	4 (1.0)
Refused	80 (9.0)	1 (0.2)
Interviewed proxy	63 (7.1)	28 (6.6)
Interviewed respondent	695 (78.1)	390 (92.2)

Demographic and socioeconomic characteristics of this sample are shown in Table 2. About one-third of our sample population was male. Median age in NGCA was slightly higher compared to GCA (73.4 and 70.4 years, respectively), but this difference was not significant. A higher proportion of persons had some higher education (complete or incomplete) in GCA than in NGCA (*P* = 0.026). More persons in NGCA were living on <$2/day (67.3%) compared to persons living in GCA (54.8%) (*P* = 0.009). Almost 85% of persons in GCA and 88% of persons in NGCA were living on less than $3/day. Nearly three-fourths (73.9%) of persons in NGCA and 67.8% of persons in GCA had been diagnosed with at least one chronic disease.

**Table 2:** Demographic and socioeconomic characteristics among persons ≥ 60 years, Ukraine, 2016

Demographic Characteristics	GCA (*N* = 758) n (%)	NGCA (*N* = 418) n (%)
Gender		
Male	266 (35.1)	140 (33.5)
Female	492 (64.9)	278 (66.5)
Age (years)		
60–70	371 (48.9)	181 (43.3)
>70	387 (51.1)	237 (56.7)
Education level		
Secondary school (incomplete)	177 (23.4)	100 (23.9)
Secondary school (complete)	177 (23.4)*	143 (34.2)*
Professional secondary education (complete or incomplete)	285 (37.6)	133 (31.8)
Higher education or above (complete or incomplete)	119 (15.7)	42 (10.1)
Housing situation		
Self-owned house or apartment (no fee)	740 (97.6)	400 (95.7)
Other	18 (2.4)	18 (4.3)
Living situation		
Living alone	238 (31.4)	123 (29.4)
Living only with another person(s) ≥ 60	308 (40.6)	172 (41.2)
Living with people < 60	212 (28.0)	123 (29.4)
Household income per capita ($/day)	*N* = 668	*N* = 339
< $2/day	366 (54.8)*	228 (67.3)*
$2–$3/day	199 (29.8)	69 (20.4)
$3–$4/day	72 (10.8)	16 (4.7)
> $4/day	31 (4.6)	26 (7.7)
Chronic disease	514 (67.8)	309 (73.9)

*P < 0.05

Table 3 shows prevalence of dependency as measured by the Katz scale and the availability of needed assistance. In GCA, 12.4% (95%CI, 9.9**–**15.4%) of persons were classified as moderately or severely dependent compared to 17.2% (95%CI, 14.2**–**20.9%) of persons in NGCA. Moderate dependency was similar in NGCA and GCA, however persons in NGCA were significantly more likely to have severe dependency than persons in GCA (*P* = 0.003). The most common dependencies were being incontinent [18.3% (GCA), 23.4% (NGCA)] and needing help bathing [14.6% (GCA), 20.3% (NGCA)]. For all assessed daily items except incontinence, persons from NGCA were significantly more likely to require assistance.

**Table 3:** Dependency and availability of needed assistance among persons ≥ 60 years, Ukraine, 2016

	GCA Total (*N* = 758)		NGCA (*N* = 418)	
	n (%)	95%CI	n (%)	95%CI
Requires help with activities				
Bathing	111 (14.6)*	12.0–17.7	85 (20.3)*	16.0–25.5
Getting dressed	62 (8.2)**	6.6–10.1	57 (13.6)**	11.1–16.6
Going to the toilet	46 (6.1)**	4.7–7.9	51 (12.2)**	9.6–5.4
Moving from bed to chair	44 (5.8)**	4.3–7.8	46 (11.0)**	8.1–14.8
Incontinent	139 (18.3)	14.5–22.9	98 (23.4)	19.3–28.2
Eating	20 (2.6)**	1.7–4.0	30 (7.2)**	4.7–10.9
Dependency^a^				
Independent	664 (87.6)*	84.6–90.1	345 (82.7)*	79.3–85.7
Moderate dependency	48 (6.3)	4.5–8.9	25 (6.0)	4.1–8.7
Severe dependency	46 (6.1)**	4.6–8.0	47 (11.3)**	8.7–14.5
Someone to assist if person needs help with 1 or more activity^b^	*N* = 186		*N* = 130	
No	31 (16.7)	11.6–23.4	32 (24.6)	15.9–36.0
Yes, but not every day	55 (29.6)**	24.5–35.3	16 (12.3)**	8.1–18.3
Yes, every day, part of the time	18 (9.7)	6.1–15.1	10 (7.7)	3.8–15.1
Yes, every day, all of the time	82 (44.1)	37.4–51.0	71 (54.6)	43.9–65.0
Don’t know	0	0	1 (0.8)	0.001–0.06

*P < 0.05; **P < 0.01

^a^ Independent = Needs help with ≤ 1 activity; Moderate dependency = Needs help with ≥ 2 and < 4 activities; Severe dependency = Needs help with ≥ 4 activities

^b^ N=Persons who reported needing help with one or more daily activity

Nearly one-fourth (24.6%) of persons needing assistance with one or more activity of daily living in NGCA and 16.7% of persons needing assistance in GCA did not have any assistance; 46.3% of those in GCA and 36.9% of those in NGCA did not have any assistance or did not have daily assistance (Table 3). About half of persons needing assistance [44.1% (GCA), 54.6% (NGCA)] had assistance every day, all of the time.

Table 4 shows the prevalence of psychological distress as assessed by the Kessler scale, which showed excellent reliability in our study sample (Cronbach’s alpha = 0.86). The prevalence was higher in NGCA, 42.5% (95%CI, 36.1**–**49.2%) than in GCA, 33.6%; (95% CI, 28.0**–**39.7%) (*P* = 0.041). The median Kessler K6 Score was four points higher in NGCA (12) than GCA (8) (*P* < 0.001).

**Table 4:** Prevalence of psychological distress among persons ≥ 60 years, Ukraine, 2016

	GCA (*N* = 711)		NGCA (*N* = 396)	
	n (%)	95%CI	n (%)	95%CI
Felt most or all of the time in past 30 days				
Nervous	286 (40.2)	35.4–45.2	162 (40.9)	34.4–47.8
Hopeless	170 (23.9) **	20.5–27.8	137 (34.6) **	28.8–40.9
Restless/Fidgety	252 (35.4)	29.4–42.0	178 (44.9)	37.8–52.3
Depressed	233 (32.8)	27.1–39.0	139 (35.1)	30.0–40.5
Everything is an effort	245 (34.5)	28.9–40.5	158 (39.9)	33.6–46.6
Worthless	151 (21.2)	17.2–25.9	89 (22.5)	18.2–27.4
Suffers from serious psychological distress^a^	239 (33.6) *	28.2–39.6	168 (42.5) *	36.4–48.9
	Mean (SD)	Median (IQR)	Mean (SD)	Median (IQR)
Kessler K6 Score	9.7 (6.7) **	8 (4–15) **	11.4 (6.8) **	12 (6–16) **

*P < 0.05; **P < 0.01

^a^ Kessler score > 12

Table 5 shows the association between psychological distress and dependency. In GCA, 65.9% (95%CI, 46.3**–**81.2%) of those who were moderately dependent and 75.0% (95%CI 55.4**–**87.9%) of those who were severely dependent experienced serious psychological distress. In NGCA, 68.2% (95%CI, 46.0**–**84.4%) of moderately dependent persons and 87.5% (95%CI, 71.1**–**95.2%) of severely dependent persons experienced serious psychological distress. Overall, 33.0% (95%CI, 28.8**–**37.5%) of persons who were independent and 75.3% (95%CI, 66.2**–**82.6%) of persons who were moderately/severely dependent reported serious psychological distress (*P* < .0001).

**Table 5:** Prevalence of serious psychological distress by dependency among persons ≥ 60 years, Ukraine, 2016

	GCA		NGCA		Overall	
	N n (%)	95%CI	N n (%)	95%CI	N n (%)	95%CI
Dependency^a^						
Independent	642		341		983	
	191 (29.8)	24.5–35.6	125 (36.7)	30.4–43.4	316 (33.0)	28.8–37.5
Moderate dependency	41		22		63	
	27 (65.9)	46.3–81.2	15 (68.2)	46.0–84.4	42 (67.0)	52.5–78.8
Severe dependency	28		32		60	
	21 (75.0)	55.4–87.9	28 (87.5)	71.1–95.2	49 (83.2)	71.4–90.8

^a^Independent = Needs help with ≤ 1 activity; Moderate dependency = Needs help with ≥ 2 and < 4 activities; Severe dependency = Needs help with ≥ 4 activities

The factors associated with serious psychological distress are shown in Table 6. Persons who were moderately or severely dependent were 5.20 (95%CI, 3.34–8.11) times more likely to suffer from serious psychological distress compared to persons who were independent. Females were 1.71 (95%CI, 1.25–2.33) times more likely than males, and persons who had been diagnosed with one or more chronic diseases were 1.54 (95%CI, 1.09–2.17) times more likely than those not diagnosed with a chronic disease to suffer from serious psychological distress.

**Table 6:** Factors associated in multivariate analyses with serious psychological distress, Ukraine, 2016

Outcome	N	Predictors	Odds Ratio	95%CI	*P*-value
Serious Psychological Distress	1105	Chronic Disease^a^	1.54	1.09–2.17	0.015
		Female^b^	1.71	1.25–2.33	0.001
		Moderately or severely dependent^c^	5.20	3.34–8.11	< 0.001

^a^Referent: Not diagnosed with a chronic disease

^b^Referent: Male

^c^Referent: Independent

## Discussion

Our study among older persons living in conflict affected areas of eastern Ukraine found unprecedented levels of serious psychological distress, much higher compared with data from older persons in other countries. For example, a study among older adults aged 60 and over in Australia using the Kessler K10 scale found 7% of persons living in the community experienced serious psychological distress compared with 15% of persons living in residential settings [16]. A survey comparing older Korean immigrants in the United States to older Koreans still living in Korea (age range 60–79 years of age) also using the Kessler K10 scale found 13% and 21% of persons experienced serious psychological distress, respectively [17]. A study of community-dwelling and institutionalized older persons in Poland (mean age 74.1 and 78.6 years, respectively) found 8.7% of community-dwelling persons and 15.8% of institutionalized persons experienced severe anxiety/depression as measured by the Euroqol 5D questionnaire [18]. According to the World Health Organization, 7% of persons over 60 years of age suffer from depression [19].

There may be an association between conflict and psychological distress in this population; conflict affected persons in previous studies also showed higher levels of mental distress among older persons. A study in Israel of adults 18 years and older exposed to missile attacks found persons > 65 years of age who had been exposed to missile attacks had higher odds of reporting elevated levels of distress than those not exposed [20]. In addition, a study among elderly Somali refugees (39% ≥ 60 years of age, 61% 50–59 years of age) in Finland found Somalis had higher levels of psychological distress as measured by the 12-item General Health Questionnaire (GHQ-12) compared with their pre-matched Finnish native controls and exposure to traumatic events was associated with higher levels of mental distress [21].

Severe economic hardship resulting from the conflict in Ukraine may also contribute to the high prevalence observed. People of lower socioeconomic status are more likely to suffer from psychological distress compared with those of higher socioeconomic status [22, 23]. Since the beginning of the conflict in Ukraine, inflation has increased; the exchange rate fell from eight Ukraine Hryvnia to one US dollar in 2013 to 26 Ukraine Hryvnia to one US dollar in 2016 without any notable increases in pensions for older persons [24]. This inflation severely impeded purchasing power and made older persons more vulnerable [9]. In our survey population, almost 85% of persons in GCA and 88% of persons in NGCA were living on less than $3/day.

There is inconsistent evidence regarding older age as a risk factor for conflict-induced mental distress. Some studies have found that older persons may be more resilient and less likely to experience post-traumatic stress disorder (PTSD) symptoms following human induced disasters [25, 26]. However, most studies reviewed found greater risk among older persons. A study comparing older (60–88 years of age) and younger (20–49 years of age) internally displaced persons (IDPs) in Croatia found that psychosomatic symptoms and disorders resulting from stressful experiences threaten older displaced persons more than younger IDPs [27]. Another study among Kosovar Albanians aged ≥15 years found those aged 65 years and older at increased risk for psychiatric morbidity [28]. A study in Afghanistan found older persons were at higher risk of experiencing mild to severe mental health problems than younger adults [29]. A cross-sectional survey conducted among IDPs in Ukraine, during the same time period as our study (March–May 2016) found older age to be significantly associated with PTSD, depression, and anxiety [10]. In addition, a study looking at the relationship between trauma and PTSD found conditional PTSD to be significantly associated with age, with the highest risk during childhood-adolescence and in persons ≥65 years of age [30].

The high prevalence of serious psychological distress highlights this population’s needs for psychological services. Even before the conflict, there was a lack of psychological services and psychiatric care in Ukraine. A study on use of mental health services in 17 countries in the WHO world mental health surveys from 2007 showed that only about 35% of persons surveyed with serious mental health disorders in Ukraine used mental health specialty services in the 12 months preceding the survey [31]. The serious mental health disorders included anxiety (agoraphobia, generalized anxiety disorder, panic disorder, post-traumatic stress disorder, social phobia, specific phobia), mood disorders (bipolar disorder, including bipolar I and II, dysthymia, major depressive disorder), and substance disorders (alcohol and drug abuse and dependence) [31]. Soviet society stigmatized mental health disorders and psychiatric diagnoses and confinement in psychiatric wards was often used as a form of political repression [32]. Ukraine is in the process of transitioning from state-run institutions to community-based services, however in 2015 the staffing of psychiatrists in hospitals was only 78%, with the greatest shortage in rural areas in district level hospitals [33].

Our study found persons living in GCA and NGCA to be quite similar in terms of demographic and socioeconomic indicators. Persons living in NGCA were more likely to be severely dependent and suffer from serious psychological distress. This could be because persons living in NGCA are more likely to be isolated than persons living in GCA. They must cross the contact line into GCA in order to collect their pensions, access markets and healthcare, and to obtain their identification documents; they are often forced to wait hours in severe weather conditions before they are allowed to cross the contact line [7]. In addition, they may feel isolated from family members who moved to GCA and may have fewer people to help them with daily activities and provide support [7].

Proper diagnosis of severe psychological distress with a diagnostic instrument is difficult, and must be done by a trained health care professional. However, in other settings where decentralized health care services are scarce, simple screening tools adapted to the local context used by trained lay community informants have been successful in increasing the utilization of mental health care services [34]. Given our finding that older persons who were moderately or severely dependent were 5.2 times more likely to experience serious psychological distress than those who were independent, the presence of serious disability could serve as one simple and sensitive screening criterion to be added to a screening tool used by community workers who work with and support older persons who could then refer those likely cases of serious psychological distress to a specialist for proper diagnosis. Over 75% of persons in our study who were moderately/severely dependent experienced serious psychological distress. Given the very high prevalence of psychological distress among the disabled, dependency may be recognized by humanitarian actors and decision makers as a possible risk factor and screening marker for psychological distress.

Our study was the only study we found on the association between psychological distress and disability in older non-veterans affected by conflict, which is a significant addition to the literature and important for humanitarian emergency programming, especially in middle-income countries with a high proportion of older persons. Several studies in other population groups have also documented an association between mental health and disability. A study in Denmark found that self-rated disability among women was strongly associated with a mental health disorder [35]. Another study in Hong Kong of persons 50 years of age and older found severe impairment in daily activity functioning was significantly associated with depressive symptoms [36]. A study among veterans with PTSD found that veterans with PTSD had poor health functioning and increased disability; 75% of respondents were 60 years of age or older [37]. In addition, a study in Afghanistan found persons who experienced war-related disability had higher odds of experiencing mental distress disorders and women with disabilities (whatever the cause of the impairment) had a higher prevalence of mental health disorders compared with non-disabled men [29]. A second study among adults in postwar Afghanistan also found disabled respondents to have lower social functioning and poorer mental health status than those who were nondisabled [38]. A study among adult Bosnian refugees in Croatia also found that disability and having depression and/or PTSD were related [39].

In addition to the association between psychological distress and disability, our study also found a significant independent association between psychological distress and chronic disease, supporting previous research. A study among older Chinese (60–79 years of age) found that poor mental health status was significantly associated with chronic disease [40]. A study of adults aged 65 and older in Britain found self-efficacy and mobility in men and health status and quality of life in women were the strongest independent predictors of psychological morbidity [41].

Our study had some limitations. We relied on self-reported data, which could have introduced bias if persons thought answering in a certain way could increase access to programs, or if they thought they would be stigmatized. It is unclear how this would affect our study. If persons thought they may have greater access to services, they may have over-reported their dependency or psychological distress; however, if persons were afraid of stigma, they may have under-reported their conditions. Some areas were inaccessible because of insecurity; persons in those areas may have experienced more psychological distress and/or disability because of closer proximity to the conflict. In addition, since this was a cross-sectional survey, we do not have information on disability and psychological distress pre-conflict, so were unable to compare the different time periods. This cross-sectional study design allowed us to establish whether an association existed between psychological distress and disability, but did not allow us to determine whether there was a causal relationship between them. Also, the lack of psychological services and psychiatric care in Ukraine pre-conflict may have contributed to the high prevalence of psychological distress found in our study [31]. With the absence of a control group who were not affected by the conflict, we could not determine the relative contribution of the conflict versus problems of psychological distress existing pre-conflict. We did not survey institutionalized persons, so population prevalence of disability and serious psychological distress may have been underestimated. There was also a high nonresponse rate for our study. However, most of the nonresponse was because households were absent or abandoned, while refusal rates were quite low. The high proportion of absent or abandoned households could have introduced some selection bias, as people who are not home could be more mobile and we do not know if they were out of the house or if they have left the conflict area.

## Conclusions

We found very high levels of serious psychological distress among older persons in both GCA and NGCA. We document a strong association between dependency and serious psychological distress, a finding not previously documented in conflict settings among non-combatants. We also found an independent association between chronic disease and psychological distress. These findings indicate that disability may serve as a good marker for mental health distress in this conflict-affected population of older people. One of the reasons mental health programming is so challenging is because persons are usually identified from self-referral only. Other barriers to mental health screening in complex emergencies include stigma and discrimination, the absence of culturally validated instruments, and lack of coordination of mental health care [42].

By 2050, in some developing countries the prevalence of disability is expected to increase four-fold as the population ages [43]. As the proportion of older persons in the population increases, an increased number of older people will be affected by conflict and humanitarian emergencies and the challenges of older persons because of social disintegration, negative social selection, and chronic dependency will need to be addressed. Health services for the disabled and persons with chronic diseases that include psychological as well as physical support could help in reducing the proportion of people needing mental health services who may not normally be identified. Development of care and support intervention models for older persons affected by conflict should include non-standard approaches such as community-based screening and care for psychological disorders.

## Additional file

Additional file 1: Assessment Questionnaire, Ukraine 2016. (PDF 145 kb)

## Abbreviations

CDC: Centers for Disease Control and Prevention
CI: Confidence Interval
GCA: Government Controlled Areas
GHQ-12: 12-item General Health Questionnaire
IDP: Internally Displaced Persons
IQR: Inter-Quartile Range
Kessler K10: Kessler Psychological Distress Scale (K10)
Kessler K6: Kessler Psychological Distress Scale (K6)
NGCA: Non-government Controlled Areas
OR: Odds Ratio
PTSD: Post-traumatic Stress Disorder
SD: Standard Deviation
UNHCR: United Nations High Commissioner for Refugees
WHO: World Health Organization
