# Supplementary material for: Serious psychological distress and disability among older persons living in conflict affected areas in eastern Ukraine: a cluster-randomized cross-sectional household survey
Source: Confl Health. 2019 Mar 25;13:10. doi: 10.1186/s13031-019-0194-0 (PMC6503356; doi:10.1186/s13031-019-0194-0)
Supplement: Supplementary file 1 — Assessment Questionnaire, Ukraine 2016. (PDF 145 kb) [file 13031_2019_194_MOESM1_ESM.pdf]

Assessment of elderly people in the Donetsk and Lugansk regions

Date of Survey [ ][ ] – [ ][ ] – [1][6]
DD MM YY

Interviewer Code [ ][ ]

Oblast [ ] → 1 = Donetsk
2 = Luhansk

Time of beginning: [ ][ ] [ ][ ]
HH MM

Cluster Number [ ][ ]

Household Number [ ][ ][ ][ ]

Respondent's Relationship to Older Person [ ] ______
1 = Self
2 = Spouse
3 = Son/Daughter/ Grandson/Granddaughter
4 = Other (Specify)

Number of people over 60 years [ ]

Code of elderly person in Household [ ]

Birth date of Older Person [ ][ ] – [ ][ ] – [ ][ ][ ][ ]
DD MM YYYY

Age of Older Person (complete years) [ ][ ]

Sex of Older Person [ ] → 1 = Male
2 = Female

Section A: Household Information

Instructions: If the person being interviewed is the older person who was selected, SAY: ***"Now I will ask you questions about the household in which you are currently living."*** *If the person interviewed is answering on behalf of another person, SAY:* ***"Now I will ask you questions about the household in which the older person is currently living. Please answer to the best of your abilities for the older person for whom this interview is about."***

**1.**	What is your/(*the older person's*) education level?	1 = Incomplete secondary school<br>2 = Complete secondary school<br>3 = Professional secondary education (technikum, uchilische)<br>4 = Incomplete higher education<br>5 = Complete higher education or above<br>9 = Don't know	[ ]
**2.**	What is the name of the settlement in which you/(*the older person*) are currently living?	Write name of settlement	____________
**3.**	In what type of housing do you/(*the older person*) currently live?	1 = House or apartment you own (no rent fee)<br>2 = Living with relatives or friends (no rent fee)<br>3 = Renting an apartment or house for a fee<br>4 = Collective center<br>5 = Other	[ ]
**4.**	Have you/(*the older person*) left your/(*their*) permanent residence?	0 = No (**Skip to Q5**)<br>1 = Yes	[ ]

	**4a**. Where was your/(*the older person's*) permanent residence located before the conflict?	1 = Donetsk city<br>2 = Luhansk city<br>3 = Donetsk Oblast<br>4 = Luhansk Oblast<br>5 = Other	☐
	**4b**. When did you/(*the older person*) leave your/(*their*) permanent residence?	☐☐-☐☐☐☐<br>**MM** **YYYY**	
**5.**	What is the total number of people, including you/(*the older person*), currently living in the household?	Write number of people	☐☐
**6.**	Are you/(*the older person*) supposed to receive a pension from the government?	0 = No (**Skip to Q7**)<br>1 = Yes<br>9 = Don't Know (**Skip to Q7**)	☐
	**6a**. If yes, did you/(*the older person*) receive your/(*their)* pension last month?	0 = No (**Skip to Q7**)<br>1 = Yes<br>9 = Don't Know (**Skip to Q7**)	☐
	**6b**. What is the amount of the pension that was received last month (Hrn)?	Write amount received.<br>(Write "9999" if don't know)	______________
**7.**	Does anyone in the household currently have a money-earning job?	0 = No<br>1 = Yes<br>9 = Don't Know	☐
**8.**	What was the total monthly income of the household last month (Hrn)?	Write income amount.<br>(Write "9999" if don't know)	______________
**9.**	Are you/(*the older person*) registered to receive additional assistance (non-pension) due to the conflict in region?	0 = No (**Skip to Section B**)<br>1 = Yes<br>9 = Don't Know (**Skip to Section B**)	☐
	**9a**. Who are you/(*the older person*) registered with to receive additional (non-pension) assistance? (state, humanitarian, volunteer organizations) (**Mark all that apply with an "X' in the box**)<br>SPECIFY, IF NECESSARY, THAT THEY CAN BE REGISTER BY THEMSELVES OR SOMEBODY COULD CALL THEM AND INFORM THEM THAT THEY WERE REGISTERED WITHOUT THEIR CONSENT.	1=Ministry of Social Policy (e.g., aid for displaced persons)<br><br>2=State Emergency Service<br><br>3= Humanitarian Organization (e.g. Red Cross, NGO). **Specify**<br><br>4= Volunteer Organization (e.g., church). **Specify**<br><br>5= Other. **Specify**<br><br>6 = Don't know	☐<br>☐<br>☐ ______________<br>☐ ______________<br>☐ ______________

Section B: Humanitarian Assistance

Instructions: If the person being interviewed is the older person who was selected, SAY: ***"Now I will ask you questions about any humanitarian assistance from any humanitarian, volunteer, or non-governmental organizations you have received since the start of the emergency."*** *If the person interviewed is answering on behalf of another person, SAY:* ***"Now I will ask you questions about any humanitarian assistance from any humanitarian, volunteer, or non-governmental organizations the older person has received since the start of the emergency. Please answer to the best of your abilities for the older person for whom this interview is about."***

**1.**	Have you/(*the older person*) received any cash or voucher/coupon (e.g. money, food, medications) assistance from non-government sources due to the conflict in region in the last 3 months (November-December-January)?	0 = No (**Skip to Q2**)<br>1 = Yes<br>9 = Don't Know (**Skip to Q2**)	☐
	**1a**. From whom have you/ *(the older person*) received cash or voucher/coupon (e.g. money, food, medications) assistance in the last 3 months (November-December-January)? (**Mark all organizations giving assistance in the last 3 months with an 'X' in the box**).<br>YOU CAN SELECT MULTIPLE ANSWERS	1 = HelpAge International<br>2 = Red Cross<br>3 = Akhmetov Foundation<br>4 = Church<br>5 = People in Need /WFP<br>6 = Mercy Corps /WFP<br>7 = ADRA/WFP<br>8 = Caritas<br>9 = Other. **Specify**<br>10 = Don't know	☐<br>☐<br>☐<br>☐<br>☐<br>☐<br>☐ ____________
	**1b**. How many times have you/(*the older person*) received cash or voucher/coupon (e.g. money, food, medications) assistance from non-government sources in the past 3 months (November-December-January)?	1 = 1 time<br>2 = 2 times<br>3 = 3 times<br>4 = >3 times<br>9 = Don't Know	☐
**2.**	Have you/(*the older person)* received any FOOD (food products) assistance from non-government sources due to the emergency in the last 3 months (November-December-January)?	0 = No (**Skip to Q2C**)<br>1 = Yes<br>9 = Don't Know (**Skip to Q2C**)	☐

	**2a**. From whom have you/*(the older person*) received FOOD (food products) assistance from in the last 3 months (November-December-January)? (**Mark all organizations giving assistance in the last 3 months with an 'X' in the box**).	1 = HelpAge International<br>2 = Red Cross<br>3 = Akhmetov Foundation<br>4 = Church<br>5 = People in Need /WFP<br>6 = Mercy Corps /WFP<br>7 = ADRA/WFP<br>8 = Caritas<br>9 = Other. **Specify**<br>10 = Don't know	☐<br>☐<br>☐<br>☐<br>☐<br>☐<br>☐ ____________
	**2b**. How many times have you/(*the older person*) received FOOD assistance from non-government sources in the past 3 months (November-December-January)? (**Skip to Q3**)	1 = 1 time<br>2 = 2 times<br>3 = 3 times<br>4 = >3 times<br>9 = Don't Know	☐
	**2c**. Why have you/(*the older person*) NOT received FOOD assistance from non-government sources in the past 3 months (November-December-January)?	1 = I do not fall under the criteria for distribution<br>2 = Not registered<br>3 = Not able to collect<br>4 = Waiting time too long<br>5 = Other (**Specify**)<br>9 = Don't know	☐ ____________
**3.**	Have you/(*the older person*) received any NON-FOOD assistance (e.g. hygiene items, detergents, blankets, household items) from non-government sources due to the emergency in the last 3 months (November-December-January)?	0 = No (**Skip to Q4**)<br>1 = Yes<br>9 = Don't Know (**Skip to Q4**)	☐
	**3a**. What NON-FOOD assistance was received? (**Mark all items received with an 'X' in the box**).	1= Hygiene items<br>2= Walking aids<br>3= Blankets/Clothing<br>4 = Items for repairing household/shelter<br>5= Other	☐<br>☐<br>☐<br>☐<br>☐

	**3b**. From whom have you/ *(the older person*) received NON-FOOD assistance in the last 3 months (November-December-January)? (**Mark all organizations giving assistance in the last 3 months with an 'X' in the box**).	1 = HelpAge International<br>2 = Red Cross<br>3 = Akhmetov Foundation<br>4 = Church<br>5 = People in Need /WFP<br>6 = Mercy Corps /WFP<br>7 = ADRA/WFP<br>8 = Caritas<br>9 = Other. **Specify**<br>10 = Don't know	☐<br>☐<br>☐<br>☐<br>☐<br>☐ ____________
	**3c**. How many times have you/(*the older person*) received NON-FOOD assistance from non-government sources in the past 3 months (November-December-January)?	1 = 1 time<br>2 = 2 times<br>3 = 3 times<br>4 = >3 times<br>9 = Don't Know	☐
**4.**	Has any organization provided transportation to you/(*the older person*) to go shopping, to the clinic, to government offices, or to collect food distribution or has any organization helped deliver assistance (food, non-food, cash or vouchers) to your/(*their*) residence in the last 3 months (November-December-January)?	0 = No (**Skip to Section C**)<br>1 = Yes<br>9 = Don't Know (**Skip to Section C**)	☐

	**4a**. Which organizations provided you/ *(the older person*) transportation to go shopping, to the clinic, to government offices, or to collect food distribution or helped deliver assistance (food, non-food, cash or vouchers) to your/(*their*) residence in the last 3 months (November-December-January)? (**Mark all organizations giving assistance in the last 3 months with an 'X' in the box**).	1 = HelpAge International<br>2 = Red Cross<br>3 = Akhmetov Foundation<br>4 = Church<br>5 = People in Need /WFP<br>6 = Mercy Corps /WFP<br>7 = ADRA/WFP<br>8 = Caritas<br>9 = Other. **Specify**<br>10 = Don't know	☐<br>☐<br>☐<br>☐<br>☐<br>☐ ____________
	**4b**. How many times have you/(*the older person*) been provided with transportation to go shopping, to the clinic, to government offices, or to collect food distribution or had assistance delivered in the past 3 months (November-December-January)?	1 = 1 time<br>2 = 2 times<br>3 = 3 times<br>4 = >3 times<br>9 = Don't Know	☐

Section C: Health Information

Instructions: If the person being interviewed is the older person who was selected, SAY: ***"Now I will ask you questions about your current health issues and medications that you are currently taking."*** *If the person interviewed is answering on behalf of another person, SAY:* ***"Now I will ask you questions about current health issues and medications that the older person is currently taking. Please answer to the best of your abilities for the older person for whom this interview is about."***

**1.**	Have you/(*the older person*) ever been diagnosed with hypertension?	0 = No (**Skip to Q2**)<br>1 = Yes<br>9 = Don't Know (**Skip to Q2**)	☐
	**1a**. Have you/(*the older person*) been told/got prescription to take hypertension medication regularly by a doctor?	0 = No (**Skip to Q2**)<br>1 = Yes<br>9 = Don't Know (**Skip to Q2**)	☐
	**1b**. Are you/(*the older person*) taking the hypertension medication regularly?	0 = No<br>1 = Yes (**Skip to Q2**)<br>9 = Don't Know (**Skip to Q2**)	☐
	**1c**. Why are you/(*the older person*) not taking the hypertension medication regularly?	1 = Can't access pharmacy<br>2 = Medication not available at pharmacy<br>3 = Medication is too expensive<br>4 = Other (**Specify**)<br>9 = Don't Know	☐ ____________

**2.**	Have you/(*the older person*) ever been diagnosed with diabetes?	0 = No (**Skip to Q3**)<br>1 = Yes<br>9 = Don't Know (**Skip to Q3**)	☐
	**2a**. Which type of diabetes?	1 = Type 1 (need insulin)<br>2 = Type 2 (treatment with medications)<br>9 = Don't Know	☐
	**2b**. Have you/(*the older person*) been told to take diabetes medication regularly by a doctor?	0 = No (**Skip to Q3**)<br>1 = Yes<br>9 = Don't Know (**Skip to Q3**)	☐
	**2c**. Are you/(*the older person*) taking the diabetes medication regularly?	0 = No<br>1 = Yes (**Skip to Q3**)<br>9 = Don't Know (**Skip to Q3**)	☐
	**2d**. Why are you/(*the older person*) not taking the diabetes medication regularly?	1 = Can't access pharmacy<br>2 = Medication not available at pharmacy<br>3 = Medication is too expensive<br>4 = Other (**Specify**)<br>9 = Don't Know	☐ ____________
**3.**	Have you/(*the older person*) ever been diagnosed with any cardiac or vascular diseases (except hypertension)? (e.g. arrhythmia, coronary heart disease, stroke/heart attack, pre-stroke/pre-heart attack condition)	0 = No (**Skip to Q4**)<br>1 = Yes<br>9 = Don't Know (**Skip to Q4**)	☐
	**3a**. Have you/(*the older person*) been told to take medication for a cardiac/vascular disease regularly by a doctor?	0 = No (**Skip to Q4**)<br>1 = Yes<br>9 = Don't Know (**Skip to Q4**)	☐
	**3b**. Are you/(*the older person*) taking the medication for cardiac/vascular disease regularly?	0 = No<br>1 = Yes (**Skip to Q4**)<br>9 = Don't Know (**Skip to Q4**)	☐
	**3c**. Why are you/(*the older person*) not taking the medication for cardiac/vascular disease regularly?	1 = Can't access pharmacy<br>2 = Medication not available at pharmacy<br>3 = Medication is too expensive<br>4 = Other (**Specify**)<br>9 = Don't Know	☐ ____________
**4.**	Have you/(*the older person*) ever been diagnosed with any chronic lung disease? (e.g., Tuberculosis, Pulmonary Failure, Asthma, BUT NOT PNEUMONIA)	0 = No (**Skip to Q5**)<br>1 = Yes<br>9 = Don't Know (**Skip to Q5**)	☐
	**4a**. Have you/(*the older person*) been told to take medication for chronic lung disease regularly by a doctor?	0 = No (**Skip to Q5**)<br>1 = Yes<br>9 = Don't Know (**Skip to Q5**)	☐
	**4b**. Are you/(*the older person*) taking the medication for chronic lung disease regularly?	0 = No<br>1 = Yes (**Skip to Q5)**<br>9 = Don't Know (**Skip to Q5**)	☐
	**4c**. Why are you/(*the older person*) not taking medication for chronic lung disease regularly?	1 = Can't access pharmacy<br>2 = Medication not available at pharmacy<br>3 = Medication is too expensive<br>4 = Other (**Specify**)<br>9 = Don't Know	☐ ____________
**5.**	Have you/(*the older person*) had a cold or another respiratory illness in the last 2 weeks? (e.g., Tonsillitis, flu, respiratory diseases)	0 = No (**Skip to Section D**)<br>1 = Yes<br>9 = Don't Know (**Skip to Section D**)	☐

	**5a**. Did you/(*the older person*) seek care from a health clinic for the cold or respiratory illness? (e.g., visit to hospital or medical center, doctor's visit to the house, but NOT JUST CONSULTATION WITH A PHARMACIST IN A PHARMACY)	0 = No<br>1 = Yes (**Skip to Section D**)<br>9 = Don't Know (**Skip to Section D**)	☐
	**5b**. Why did you/(*the older person*) NOT seek care from a clinic for the cold or respiratory illness?	1 = Can't access the clinic (e.g., destroyed, closed, far)<br>2 = Clinic too expensive<br>3 = Didn't feel it was necessary<br>4 = Other (**Specify**)<br>9 = Don't Know	☐ ____________

Section D: Nutrition

Instructions: If the person being interviewed is the older person who was selected, SAY: ***"Now I will ask you questions about what types of foods you are eating."*** *If the person interviewed is answering on behalf of another person, SAY:* ***"Now I will ask you questions about what types of foods the older person is eating. Please answer to the best of your abilities for the older person for whom this interview is about."***

Ask **"Yesterday, what did you/(*the older person*) eat or drink throughout the day? Include all meals and snacks starting with the first thing you/(*the older person*) ate in the morning when you/(*the older person*) woke up and ending with the last thing you/(*the older person*) ate at night before bed."** *Record all foods the respondent mentions. When composite dishes are mentioned, ask for the list of ingredients. When the respondent has finished, probe for meals and snacks not mentioned.*

Breakfast	Snack	Lunch	Snack	Dinner	Snack

1. *When the respondent recall is complete, fill in the food groups based on the information recorded above. For any food group not mentioned, ask the respondent if a food item from this group was consumed. Record a response for all foods. Do not leave any boxes blank.*

No	Type of Food	Response	
**a.**	Cereals (bread, wheat, rice, buckwheat, corn, oats, semolina, muesli, other breakfast cereal)	☐	0 = No<br>1 = Yes<br>9 = Don't Know
**b.**	White/yellow potatoes and turnips	☐	
**c.**	Dark leafy vegetables (broccoli, spinach, lettuce, watercress)	☐	
**d.**	Vitamin A rich vegetables (carrot, pumpkin, red sweet pepper, sweet potato, squash), incl. canned	☐	
**e.**	Vitamin A rich fruits (peach, apricots, persimmon, melon, mango, papaya), incl. canned	☐	
**f.**	Other vegetables (including mushrooms)	☐	
**g.**	Other fruits, incl. canned and dried fruits	☐	
**h.**	Eggs	☐	
**i.**	Organ meats, fresh or canned (liver, kidney, lungs, heart)	☐	
**j.**	Meat, fresh or canned (beef, lamb, rabbit, chicken, sausages)	☐	
**k.**	Fish and seafood (fresh or canned)	☐	
**l.**	Milk and milk products (sour milk, animal milk, kefir, yoghurt, prostokvasha, cottage cheese (tvorog), cheese (syr))	☐	
**m.**	Peas, beans, lentils, chickpeas, soybeans, nuts, or seeds (any foods made from these)	☐	

2. How many meals and snacks did you/(*the older person*) eat yesterday? ☐☐
(Write "99" if Don't Know)

3. Ask **"How many DAYS (NOT TIMES) during the last week (last 7 days) did you/(*the older person*) eat the following foods?"**
Ask for all foods. Record number of days in box. Do not leave any boxes blank. (Write "9" if Don't Know)

No	Type of Food	Response	
**a.**	Cereals (bread, wheat, rice, buckwheat, corn, oats, semolina, muesli, other breakfast cereal)	☐	Record number of days<br>0 = Did not eat<br>9 = Don't Know
**b.**	White/yellow potatoes and turnips	☐	
**c.**	Vegetables (all, incl. mushrooms)	☐	
**d.**	Fruits (all)	☐	
**e.**	Eggs	☐	
**f.**	Meats, organ meats, poultry, fish and seafood	☐	
**g.**	Milk and milk products (sour milk, animal milk, kefir, yoghurt, prostokvasha, cottage cheese (tvorog), cheese (syr))	☐	
**h.**	Peas, beans, lentils, chickpeas, soybeans, nuts, or seeds (any foods made from these)	☐	

Instructions: If the person being interviewed is the older person who was selected, SAY: ***"Now I will ask you questions about times you or other members of your household may have been hungry in the past 30 days."*** *If the person interviewed is answering on behalf of another person, SAY:* ***"Now I will ask you questions about times the older person or other members of their household may have been hungry in the past 30 days. Please answer to the best of your abilities for the older person for whom this interview is about."***

**4.**	In the past 30 days, how many times was there **no food to eat of any kind** in your/(*the older person's*) house because of lack of resources to get food?	0 = 0 times<br>1 = 1-2 times<br>2 = 3-10 times<br>3 = > 10 times<br>9 = Don't know	☐
**5.**	In the past 30 days, how many times did you/(*the older person*) or any household member **go to sleep at night hungry**?	0 = 0 times<br>1 = 1-2 times<br>2 = 3-10 times<br>3 = > 10 times<br>9 = Don't know	☐
**6.**	In the past 30 days, how many times did you/(*the older person*) or any household member go **a whole day and night without eating anything at all** because there was not enough food?	0 = 0 times<br>1 = 1-2 times<br>2 = 3-10 times<br>3 = > 10 times<br>9 = Don't know	☐

Section E: Activities of Daily Living

Instructions: If the person being interviewed is the older person who was selected, SAY: ***"Now I will ask you questions about your activities of daily living."*** *If the person interviewed is answering on behalf of another person, SAY:* ***"Now I will ask you questions about the older person's activities of daily living. Please answer to the best of your abilities for the older person for whom this interview is about."***

**1.**	Do you/(*the older person*) need help with bathing more than one part of your body or getting in and out of the tub or shower?	0 = No<br>1 = Yes<br>9 = Don't know	☐
**2.**	Do you/(*the older person*) need help getting dressed partially or completely (not including tying of shoes)?	0 = No<br>1 = Yes<br>9 = Don't know	☐
**3.**	Do you/(*the older person*) need help going to the toilet or cleaning yourself after using the toilet?	0 = No<br>1 = Yes<br>9 = Don't know	☐
**4.**	Do you/(*the older person*) need someone (I.E. NOT A WALKING AID BUT PERSON) to help you move from a bed to a chair?	0 = No<br>1 = Yes<br>9 = Don't know	☐
**5.**	Do you/(*the older person*) leak urine or feces, including when you cough, laugh, or sneeze?	0 = No<br>1 = Yes<br>9 = Don't know	☐
**6.**	Do you/(*the older person*) need partial or total help with eating? (NOT COOKING BUT, E.G., HOLD CUTLERY)	0 = No<br>1 = Yes<br>9 = Don't know	☐

**7.**	Is someone taking care of you/(*the older person*) or helping with everyday activities such as shopping, cooking, bathing, and dressing?	0 = No<br>1 = Yes, but not every day<br>2 = Yes, every day, but not the whole day<br>3 = Yes, every day, all of the time<br>9 = Don't know	☐

Section F: Psychological Condition

Instructions: Only ask these questions if the person being interviewed is the older person who was selected, SAY: ***"Now I will ask you questions about how you have been feeling in the past 30 days."***

**1.**	During the past 30 days, about how often did you feel nervous? Would you say all of the time, most of the time, some of the time, a little of the time, or none of the time?	0 = None of the time<br>1 = A little of the time<br>2 = Some of the time<br>3 = Most of the time<br>4 = All of the time<br>6 = Refused<br>9 = Don't know	☐
**2.**	During the past 30 days, about how often did you feel hopeless? Would you say all of the time, most of the time, some of the time, a little of the time, or none of the time?	0 = None of the time<br>1 = A little of the time<br>2 = Some of the time<br>3 = Most of the time<br>4 = All of the time<br>6 = Refused<br>9 = Don't know	☐
**3.**	During the past 30 days, about how often did you feel restless or fidgety? Would you say all of the time, most of the time, some of the time, a little of the time, or none of the time?	0 = None of the time<br>1 = A little of the time<br>2 = Some of the time<br>3 = Most of the time<br>4 = All of the time<br>6 = Refused<br>9 = Don't know	☐
**4.**	During the past 30 days, about how often did you feel so depressed that nothing could cheer you up? Would you say all of the time, most of the time, some of the time, a little of the time, or none of the time?	0 = None of the time<br>1 = A little of the time<br>2 = Some of the time<br>3 = Most of the time<br>4 = All of the time<br>6 = Refused<br>9 = Don't know	☐
**5.**	During the past 30 days, about how often did you feel that everything was an effort? Would you say all of the time, most of the time, some of the time, a little of the time, or none of the time?	0 = None of the time<br>1 = A little of the time<br>2 = Some of the time<br>3 = Most of the time<br>4 = All of the time<br>6 = Refused<br>9 = Don't know	☐
**6.**	During the past 30 days, about how often did you feel worthless? Would you say all of the time, most of the time, some of the time, a little of the time, or none of the time?	0 = None of the time<br>1 = A little of the time<br>2 = Some of the time<br>3 = Most of the time<br>4 = All of the time<br>6 = Refused<br>9 = Don't know	☐

Section G: Measurements

PLEASE NOTE, WHEN YOU MEASURE RESPONDENT'S WEIGHT, REPEAT THE PROCEDURE THREE TIMES AND WRITE DOWN THE RESULTS IN THE BOXES ACCORDINGLY: WEIGHT 1 (KG), WEIGHT 2 (KG), WEIGHT 3 (KG). EACH TIME ASK THE RESPONDENT TO STEP ON THE SCALES, WEIGH THEM, NOTE THE RESULT AND ASK THEM TO COMPLETELY STEP OFF THE SCALES FOR FEW SECONDS.

IF RESPONDENT REFUSE TO PARTICIPATE IN ANY OF THE MEASUREMENTS, PLEASE FILL EVERY EMPTY CELL WITH «6». E.G., IF RESPONDENT REFUSES TO MEASURE MUAC, IT IS NECESSARY TO WRITE IN THE APPROPRIATE COLUMN "666.6".
IF YOU CAN NOT MAKE ANY MEASUREMENTS BECAUSE OF THE HEALTH CONDITION OF THE RESPONDENT (E.G., BEDRIDDEN PATIENTS), PLEASE FILL EVERY EMPTY CELL WITH «9». E.G, IF THE RESPONDENT CAN NOT BE WEIGHED, BECAUSE HE CAN NOT GET UP FROM THE BED, IT IS NECESSARY TO WRITE IN THE APPROPRIATE COLUMN "999.9".

Measure MUAC (mm) ☐☐☐

Arm Demi-Span (cm) ☐☐☐.☐

Weight 1 (kg) ☐☐☐.☐

Weight 2 (kg) ☐☐☐.☐

Weight 3 (kg) ☐☐☐.☐

Time Visit Completed ☐☐ ☐☐
HH **MM**

Comments (e.g., reason why it is impossible to do measurements with the respondent):

____
____
____
____
____
